# Aurasperone A Inhibits SARS CoV-2 In Vitro: An Integrated In Vitro and In Silico Study

**DOI:** 10.3390/md20030179

**Published:** 2022-02-28

**Authors:** Mai H. ElNaggar, Ghada M. Abdelwahab, Omnia Kutkat, Mohamed GabAllah, Mohamed A. Ali, Mohamed E. A. El-Metwally, Ahmed M. Sayed, Usama Ramadan Abdelmohsen, Ashraf T. Khalil

**Affiliations:** 1 Department of Pharmacognosy, Faculty of Pharmacy, Kafrelsheikh University, Kafrelsheikh 33516, Egypt; 2 Department of Pharmacognosy, Faculty of Pharmacy, Mansoura University, Mansoura 35516, Egypt; prof_kali@yahoo.com; 3 Department of Pharmacognosy, Faculty of Pharmacy, Horus University, Damietta 34518, Egypt; 4 Center of Scientific Excellence for Influenza Viruses, National Research Centre, Giza 12622, Egypt; omniakutkat@gmail.com (O.K.); gaballah09@gmail.com (M.G.); mohamedahmedali2004@yahoo.com (M.A.A.); 5 National Institute of Oceanography and Fisheries (NIOF), Hurghada 54511, Egypt; metwally.niof@yahoo.com; 6 Department of Pharmacognosy, Faculty of Pharmacy, Nahda University, Beni-Suef 62513, Egypt; ahmed.mohamed.sayed@nub.edu.eg; 7 Department of Pharmacognosy, Faculty of Pharmacy, Almaaqal University, Basra 61014, Iraq; 8 Department of Pharmacognosy, Faculty of Pharmacy, Minia University, Minia 61519, Egypt; usama.ramadan@mu.edu.eg; 9 Department of Pharmacognosy, Faculty of Pharmacy, Deraya University, New Minia 61111, Egypt

**Keywords:** *Aspergillus niger*, Phallusia nigra, Rubasperone B, Aurasperone A, SARS CoV-2, M^pro^, in silico, antiviral

## Abstract

Several natural products recovered from a marine-derived *Aspergillus niger* were tested for their inhibitory activity against SARS CoV-2 in vitro. Aurasperone A (**3**) was found to inhibit SARS CoV-2 efficiently (IC_50_ = 12.25 µM) with comparable activity with the positive control remdesivir (IC_50_ = 10.11 µM). Aurasperone A exerted minimal cytotoxicity on Vero E6 cells (CC_50_ = 32.36 mM, SI = 2641.5) and it was found to be much safer than remdesivir (CC_50_ = 415.22 µM, SI = 41.07). To putatively highlight its molecular target, aurasperone A was subjected to molecular docking against several key-viral protein targets followed by a series of molecular dynamics-based in silico experiments that suggested M^pro^ to be its primary viral protein target. More potent anti-SARS CoV-2 M^pro^ inhibitors can be developed according to our findings presented in the present investigation.

## 1. Introduction

Infection with the severe respiratory disease caused by SARS-CoV-2 virus emerged at the end of 2019. COVID-19 disease has become one of the worst pandemics in the modern age [1]. It has resulted in tragic effects on the economy, social life, and health care systems worldwide [2]. Despite the expectations of controlling it through the adoption of highly effective vaccinations, it is difficult to predict its evolutionary future, given the explosion in outbreaks of evasive variants [3]. The threats of emergence of more genetic variants with higher virulence and the increased rate of viral resistance to approved drugs is driving a continuous need for search for effective antiviral drugs against SARS-CoV-2. 

Marine-associated fungi represent a powerful source of antiviral secondary metabolites [4,5,6,7]. *Aspergillus* species have been reported to be significant suppliers of antiviral therapeutics [4,5]. *Aspergillus niger* is one of the most important and widely spread fungal species [8]. It is characterized by its ability to produce variable classes of natural products with different biological activities [8,9]. It also represents a rich source for unpredicted and novel secondary metabolites due to the presence of cryptic biosynthetic gene clusters in its genome [8,10]. Naphthopyrones and their dimers are among the most important secondary metabolites produced by *A. niger*. They are thought to be defense metabolites formed under stressful conditions to protect fungi from predators through a non-toxic mechanism [11]. They are reported to have antitumor and antimicrobial activities and to be inhibitors for several enzymes such as xanthine oxidase, HIV-1 integrase, and *Taq* DNA polymerase [12]. Moreover, aspernigrin alkaloid produced by *A. niger* has been reported to exert anti-HIV-1 activity [13] and to display neuroprotective and antiproliferative effect against some cancer cell lines [14]. The importance of the fungal secondary metabolites and the variation in their biological activities motivated us to continue our research work on *A. niger*.

In this study, we report the isolation of one naphthopyrone from *A. niger* that was previously isolated from the Red Sea tunicate, *Phallusia nigra* [15]. It was identified as rubasperone B (**6**). We investigated the antiviral activity of the isolated compound against SARS-CoV-2 together with other compounds isolated in our previous study [15] including four naphthopyrones, namely, flavasperone (**1**), rubrofusarin B (**2**), aurasperone A (**3**), fonsecinone A (**4**) and aspernigrin A alkaloid (**5**). Several in silico techniques, including molecular docking, molecular dynamics simulations (MDS), and end-state thermodynamics, are widely used as valuable tools for explaining the biological activities and the binding ability of the tested molecules to important therapeutic targets [16,17,18]. SARS-CoV-2 has two proteases, the main protease (M^pro^), and the papain-like protease (PL^pro^), which are essential for processing the viral non-functional proteins, and they are among the most important therapeutic targets used for the treatment of COVID-19 [19,20,21]. SARS-CoV-2 helicase and RNA-dependent RNA polymerase (RdRp) are critical enzymes involved in the viral genome replication and repair. That is why they are considered to be attractive targets for SARS-CoV-2 antiviral drugs [22,23,24]. The viral spike protein plays an important role in the viral entry to the host cell by interaction with the angiotensin-converting enzyme 2 (ACE2) receptors and interfering with this interaction would result in inhibition of the viral infection [18,25]. Several in silico studies have been carried on these targets using molecular docking and MDS for the development of SARS-CoV-2 inhibitors [18,26,27]. Therefore, an in silico docking study of the tested molecules on these therapeutic targets was performed to investigate their mode of action. Subsequently, a series of 150-ns-long MDS experiments were conducted to highlight the most probable target that might be involved in the anti-SARS CoV-2 activity and to study the mode of interaction of the active compound(s) with the corresponding protein target(s).

## 2. Results and Discussion

*Aspergillus niger* was isolated from the Red Sea tunicate *Phallusia nigra* collected from the coastal coral reef of the Red Sea in Hurghada, Egypt in June 2017. Wheat solid medium of *A. niger* was extracted using EtOAc after three weeks of fermentation as described previously [15].

### 2.1. Purification and Characterization of Compounds **1–6**

**Figure 1:**
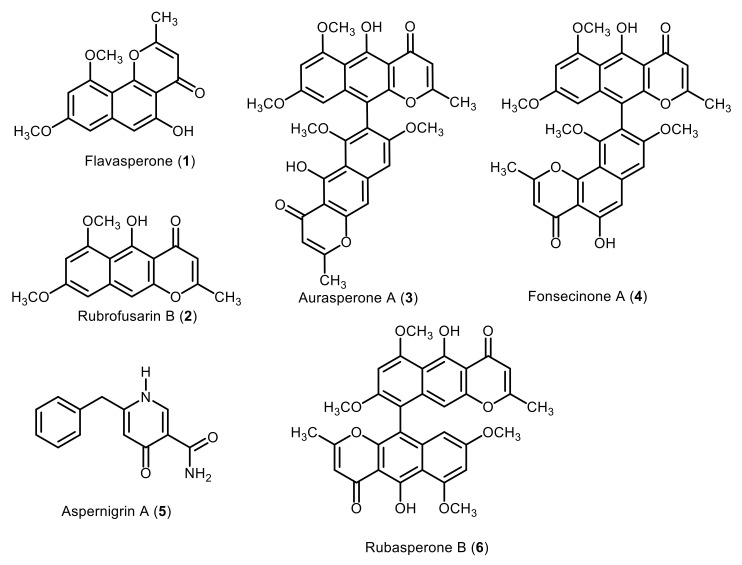
Chemical structures of the compounds (**1**–**6**) isolated from *Aspergillus niger* culture.

Compounds **1**–**5** were isolated, and their characterized structures were previously reported [15]. These compounds were found to be flavasperone (**1**), rubrofusarin B (**2**), aurasperone A (**3**), fonsecinone A (**4**) and aspernigrin A alkaloid (**5**), (Figure 1). 

Further chromatographic separation of the sub-fractions obtained from the EtOAc extract of *A. niger* resulted in the isolation of another naphtopyrone. The structure of compound **6** was elucidated using its ESI^+^ mass spectrum (Figure S1), which showed a protonated molecular ion peak [M + H]^+^ at *m/z* 571.3 assigned to the molecular formula C_32_H_27_O_10_, calculated 571.1604. The obtained molecular formula indicated 20 degrees of unsaturation suggesting the presence of two units of naphthopyrones. The ^1^H and ^13^C NMR spectra (Figures S2–S5) revealed the presence of four oxygenated and two non-oxygenated methyl groups and confirmed the presence of the two naphthopyrone units. The position of the methoxy groups and the linkage between the two naphthopyrone nucleuses was determined using HSQC and HMBC correlations (Figures S6–S8). Accordingly, the structure of compound **6** was characterized as the dimeric naphthopyrone, rubasperone B (Figure 1). The spectral data agreed with those reported in the literature [28]. This is the second report of this compound and the first time to be isolated from *A. niger*. 

### 2.2. In Vitro Antiviral Activity

**Figure 2:**
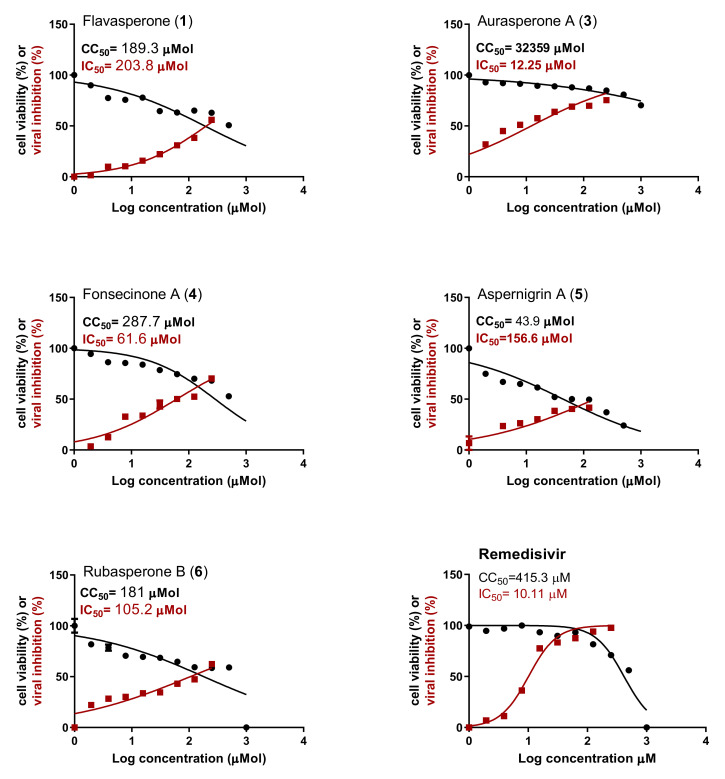
Cytotoxicity assay of the tested compounds in Vero E6 cells. The cytotoxicity of the tested compounds and remdesivir based on the dose–response was determined using MTT. The 50% cytotoxic concentration (CC_50_) was calculated for each compound using nonlinear regression analysis of GraphPad Prism software (version 5.01). Inhibitory concentration 50% (IC_50_) values were calculated using nonlinear regression analysis of GraphPad Prism software (version 5.01) by plotting log inhibitor versus normalized response (variable slope).

The antiviral activity of the isolated compounds was investigated using Vero E6 cells infected with SARS-CoV-2 virus (hCoV-19/Egypt/NRC-03/2020 (Accession Number on GSAID: EPI_ISL_430820)). Aurasperone A (**3**) showed the highest antiviral activity among the tested compounds (Figure 2). It showed potent antiviral activity against SARS-CoV-2 with IC_50_ = 12.25 µM that is comparable to the activity of the antiviral drug remdesivir (IC_50_ = 10.11 µM). Moreover, aurasperone A (3) was found to be safe to the Vero E6 cells with CC_50_ = 32.36 mM. It showed a selectivity index (SI = CC_50_/IC_50_= 2641.5) that is much higher than that of the positive control, remdesivir (SI = 41.07). These results indicate that aurasperone A could represent a promising antiviral candidate drug with lower side effects to the human than remdesivir. Fonsecinone A (**4**) showed medium antiviral activity against SARS-CoV-2 (IC_50_ = 61.06 µM) with SI = 4.7. The obtained results indicate that dimeric naphthopyrones shows better antiviral activity than the tested alkaloid (aspernigrin A, **5**) and the monomeric naphthopyrone (flavasperone, **1**). There was not enough amount of compound **2** (rubrofusarin B) to be tested in vitro. The obtained results also indicate that the type of connection between the two naphthopyrone units greatly affects their antiviral activity. The 10,7’ connected linear naphthopyrone (aurasperone A) showed higher activity than fonsecinone A and rubasperone B, with different types of connections.

### 2.3. Docking Study

**Figure 3:**
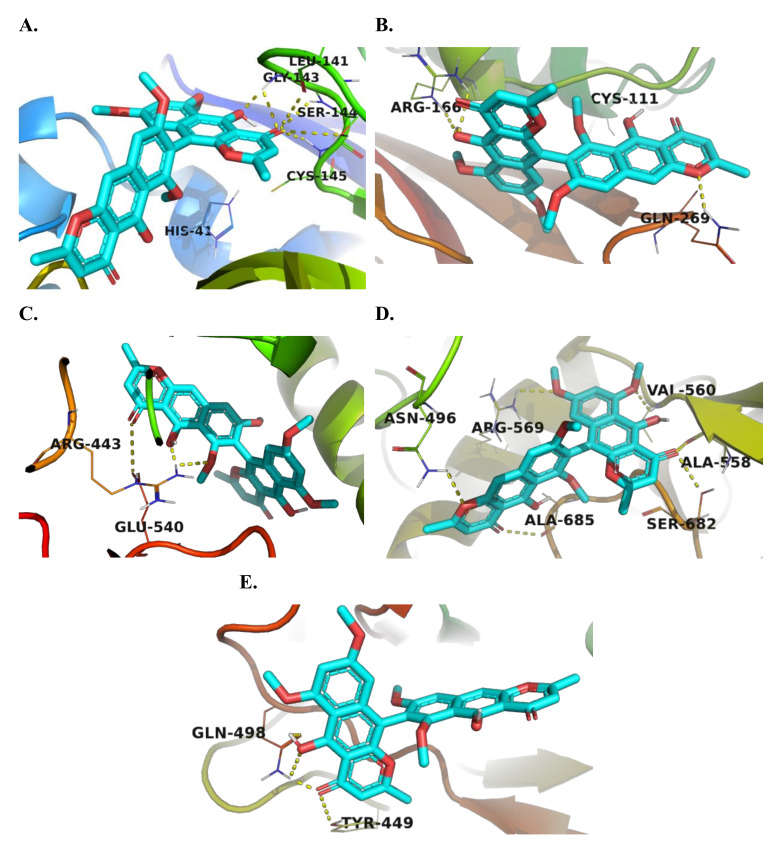
3D molecular model representation of the most active compound (aurasperone A (**3**)) binding within the active site of: (**A**) M^pro^; (**B)** PL^pro^; (**C**) RNA helicase; (**D**) RdRp; (**E**) viral spike protein, showing the amino acid residues involved in the interaction.

In silico docking study was performed to explore the mechanism of action of the tested compounds and their ability to bind to the major therapeutic targets involved in the replication of SARS-CoV-2. The dimeric naphthopyrones aurasperone A (**3**), fonsecinone A (**4**), and rubasperone B (**7**) showed the highest binding affinity among all tested targets. Their binding affinity was also found to be higher than that of the co-crystalized ligands. Aurasperone A (**3**) showed high docking scores (−8.1, −8.0, and −7.8 kcal/mol, Table 1) towards the main protease (M^pro^), helicase, and RdRp respectively. Visualization of the best docking pose of aurasperone A (**3**) against M^pro^ showed the formation of H-bond with Cys-145 amino acid residue (Figure 3A and Figure S9A), which is essential for the enzyme’s catalytic protease activity [29], in addition to Gly 143 and Ser-144 amino acids present in the active site of the enzyme. Aurasperone A (3) interacted with the ADP site of SARS-CoV-2 RNA helicase through hydrogen bonds with Arg-443 and Glu-540 residues and hydrophobic interaction with several other amino acid residue, as shown in Figure S9C [30]. It also interacted with Asn-496, Ala-558, Val-560, Arg-569, and Ala-685 residues, which are involved in the RdRp interaction with the viral RNA (Figure 3D and Figure S9D) [31]. Aurasperone A (**3**) showed reasonable binding towards PL^pro^ and the viral spike protein with docking scores of −7.4 and −7.0 kcal/mol, respectively. It interacted with Arg-166 and Gln-269 residues in the active site of PL^pro^ through hydrogen bonding and with several other residues through hydrophobic interaction. It formed a hydrogen bond with Gln-498 residue in the receptor-binding domain (RBD) of the S1 subunit of the spike protein and interacted with Arg-403, Lys-417, Tyr-449, Tyr-453, Ser-494, Gly 496, Phe-497, Asn-501, and Tyr-503 residues, which are involved in the interaction with the human ACE2 receptor through hydrophobic interaction [32].

### 2.4. Molecular Dynamics Simulation

As concluded from the previous findings, aurasperone A (**3**) was the most active compound against SARS CoV-2 in vitro. According to the docking experiments, the structure of this compound got comparable binding scores ranging from −7.0 to −8.1 kcal/mol against the five tested protein structures (Table 1). To highlight the protein targets that putatively are involved in the anti-SARS CoV-2 activity of this compound, we estimated the absolute binding free energy (Δ*G*_binding_) of aurasperone A (3) with each protein using the molecular dynamics simulation-based free energy perturbation (FEP) method [36]. Aurasperone A (**3**) was found to have the lowest Δ*G*_binding_ value with M^pro^ (−9.8 kcal/mol) indicating very good affinity to this protein target. Estimated Δ*G*_binding_ values of this compound with the remaining protein structures were lower than −6.1 kcal/mol, indicating that they are likely not involved in the inhibition mode of aurasperone A (**3**) (Δ*G*_binding_ = −6.1, −5.3, −4.6, −4.1 kcal/mol for PL^pro^, helicase, RdRp, S-protein, respectively).

Consequently, we selected M^pro^ for a further molecular dynamics-based investigation to find out how it can interact with aurasperone A (**3**). We subjected the aurasperone A-M^pro^ complex to 150 ns MDS in two independent experiments. Additionally, we built the simulation system using M^pro^ in its functioning dimeric form, trying to make the MDS experiments as realistic as we could.

**Figure 4:**
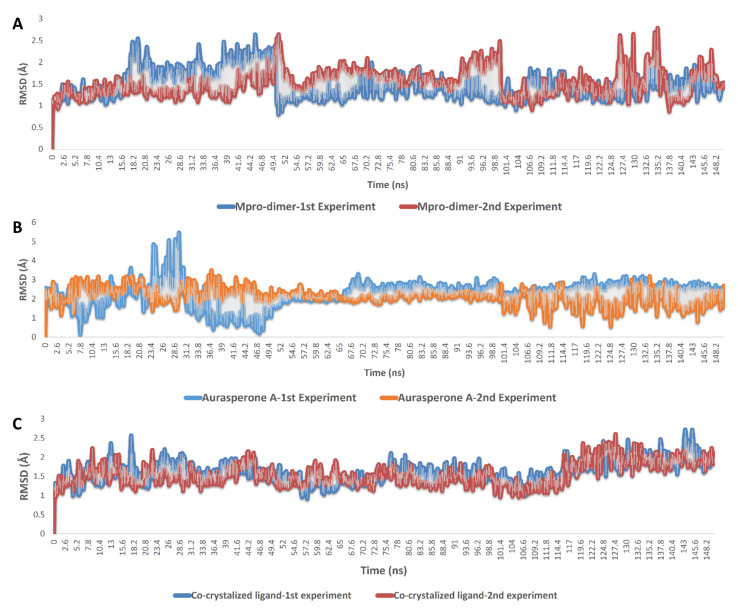
RMSDs of dimeric M^pro^ (**A**) and aurasperone A inside the M^pro^’s active site (**B**) along with the previously reported co-crystalized inhibitor YD1 (**C**) over 150 ns. The MDS experiment was carried out twice.

**Figure 5:**
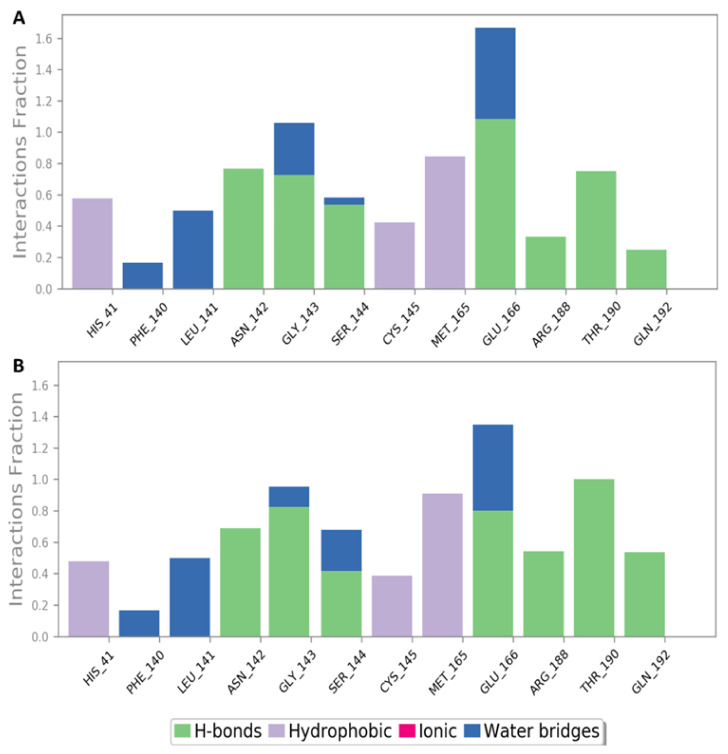
M^pro^-aurasperone A contacts over two independent 150 ns MDS experiments, (**A**) and (**B**) respectively.

As shown in Figure 4 and Figure 5, the results obtained from two 150 ns MDS experiments were convergent and comparable with that of YD1, the previously reported non-covalent M^pro^ inhibitor. Average RMSD values of aurasperone A (**3**) inside the M^pro^’s active site were slightly fluctuating during the first 50 ns, and then started to be steady till the end of the simulation. Aurasperone A (**3**) reached its maximum deviation from the starting binding orientation at 28.6 ns (RMSD = 5.3 Å), and the overall deviation until the end of simulation was ~2.9 Å.

Aurasperone A interactions inside the active site were almost the same during the two MDS experiments and were also convergent to that of the docking experiments (Figure 3A, Figure S9A and Figure 5). The compound was able to keep its H-bonding with GLY 143, and SER-144, and establish new significant H-bonds with ASN-142, GLU-166, ARG-188, THR-190, and GLN-192. In addition, there were several important water bridges with LEU-141, GLY 143, and GLU-166. Regarding hydrophobic interactions, they were kept over the MDS, in particular those with HIS-41, CYS-145, and MET-165.

From the previous in silico-based findings, we can conclude that aurasperone A (**3**) is a potential anti-SARS CoV-2 natural product that acted by inhibiting the catalytic activity of M^pro^. In silico-based experiments revealed its superior affinity and interaction with M^pro^ over other suggested molecular targets, and hence, this structural information can be used in the future to develop more potent M^pro^ inhibitors based on the structure of aurasperone A (**3**). It worth noting that aurasperone A has a good calculated druglike properties according to Lipinski and Veber’s rules.

## 3. Materials and Methods

### 3.1. General Experimental Procedures

^1^H (400 MHz) and ^13^C (100 MHz) NMR spectra and heteronuclear correlations (HSQC, and HMBC) were obtained in CDCl_3_ with TMS as internal standard on BRUKER Avance III spectrometer. Mass spectra was obtained by Advion compact mass spectrometer (CMS) Ithaca, NY, USA) using TLC mass interface and ESI negative and positive ionization modes. Chromatographic separation was performed using normal phase silica gel G 60-230 mesh (Merck, Darmstadt, Germany). The purity of the isolated compounds was observed using silica gel 60 GF_254_ TLC (20 × 20 cm, 0.2 mm thick) pre-coated on aluminum sheets (Merck, Darmstadt, Germany) and the obtained TLC spots were visualized using an ultraviolet lamp (Desaga, Wiesloch, Germany) at 254 and/or 365 nm or using vanillin/sulfuric acid spray reagent. The used solvents were purchased from commercial suppliers and distilled before use.

### 3.2. The Used Fungal Isolate, Fermentation, and Extract Preparation

The used *Aspergillus niger* strain (GenBank accession No.LC582533) was previously isolated from the Red Sea tunicate *Phallusia nigra* and identified by DNA sequencing of the Internal Transcript Spacer regions using the universal fungal primers ITS1 and ITS4 [15]. *A. niger* was fermented in 1L *Erlynmeyer* flasks containing wheat and aged sea water for three weeks then extracted using EtOAc as described previously [15].

### 3.3. Compounds Isolation and Characterization

The dried defatted EtOAc extract (6 g) obtained from *A. niger* culture was fractionated over silica gel column chromatography (CC) using a gradient of EtOAc in dichloromethane (DCM) with increasing polarity. Compounds **1** and **2** were isolated from the first sub-fraction, eluted with 100% DCM, by subjecting it to silica gel CC using isocratic elution with petroleum ether: DCM (2:8) solvent system. Compounds **3** and **4** were obtained from sub-fraction 3, eluted with 10% EtOAc in DCM, using silica gel CC then further purification with Sephadex LH-20 as previously reported [15]. Compound **5** was purified by recrystallization of sub-fraction 15, eluted with 100% EtOAc [15]. While compound **6** is isolated from sub-fraction 2.

#### Isolation and Purification of Compound **6**

TLC of sub-fraction 2 (128 mg, eluted with 5% EtOAc in DCM) showed the presence of compounds **3** and **4** in addition to other minor less polar compound. Sub-fraction 2 was subjected to silica gel CC using gradually increasing polarity of EtOAc in DCM. Sub-fraction 8 (50 mg, eluted with 4% EtOAc in DCM) was re-chromatographed over silica gel CC using gradually increasing polarity of EtOAc in DCM to afford compound **6** (5 mg, eluted with 3.5% EtOAc in DCM, yellow powder, Rf: 0.41 (100% DCM); ^1^H NMR (400 MHz, CDCl_3_) δ_H_ 15.26 (s, 1H), 15.15 (s, 1H), 6.69 (s, 1H), 6.39 (d, *J* = 2.3 Hz, 1H), 6.31 (s, 1H), 6.01 (d, *J* = 2.3 Hz, 1H), 5.96 (s, 1H), 5.92 (s, 1H), 4.15 (s, 3H), 4.01 (s, 3H), 3.79 (s, 3H), 3.48 (s, 3H), 2.20 (s, 3H), 2.01 (s, 3H); ^13^C NMR (101 MHz, CDCl_3_) δ_C_ 184.70, 184.31, 167.78, 167.69, 163.36, 162.74, 161.68, 161.52, 161.19, 159.34, 153.31, 151.13, 140.89, 140.01, 108.76, 108.64, 108.56, 107.26, 107.13, 106.82, 104.46, 104.16, 99.06, 97.08, 96.30, 92.64, 56.36, 56.27, 56.18, 55.14, 20.65, 20.62; ESI^+^-Ms *m*/*z* 571.3 [M + H]^+^ (calcd for C_32_H_27_O_10_,: 571.1604). 

### 3.4. Antiviral Activity

#### 3.4.1. MTT Cytotoxicity Assay 

To assess the half maximal cytotoxic concentration (CC_50_), stock solutions of the test compounds were prepared in 10% DMSO in ddH_2_O and diluted further to the working solutions with DMEM. The cytotoxic activity of the extracts was tested in Vero E6 cells by using the 3-(4,5-dimethylthiazol-2-yl)-2,5-diphenyltetrazolium bromide (MTT) method with minor modifications. Briefly, the cells were seeded in 96-well plates (100 µL/well at a density of 3 × 105 cells/mL) and incubated for 24 h at 37 °C in 5%CO_2_. After 24 h, cells were treated with various concentrations of the tested compounds in triplicates. 24 h later, the supernatant was discarded, and cell monolayers were washed with sterile 1x phosphate buffer saline (PBS) 3 times and MTT solution (20 µL of 5 mg/mL stock solution) was add to each well and incubated at 37 °C for 4 h followed by medium aspiration. In each well, the formed formazan crystals were dissolved with 200 µL of acidified isopropanol (0.04 M HCl in absolute isopropanol = 0.073 mL HCL in 50 mL isopropanol). Absorbance of formazan solutions was measured at λ max 540 nm with 620 nm as a reference wavelength using a multi-well plate reader. The percentage of cytotoxicity compared to the untreated cells was determined with the following equation. The plot of % cytotoxicity versus sample concentration was used to calculate the concentration which exhibited 50% cytotoxicity (CC_50_).

% cytotoxicity = (absorbance of cells without treatment − absorbance of cells with treatment)/(absorbance of cells without treatment) × 100(1)

#### 3.4.2. Inhibitory Concentration 50 (IC_50_) Determination 

In 96-well tissue culture plates, 2.4 × 104 Vero E6 cells were distributed in each well and incubated overnight at a humidified 37 °C incubator under 5%CO_2_ condition. The cell monolayers were then washed once with 1× PBS and subjected to virus adsorption (hCoV-19/Egypt/NRC-03/2020 (Accession Number on GSAID: EPI_ISL_430820)) for 1 h at room temperature (RT). The cell monolayers were further overlaid with 100 μL of DMEM containing varying concentrations of the test compounds. Following incubation at 37 °C in 5% CO_2_ incubator for 72 h, the cells were fixed with 100 μL of 4% paraformaldehyde for 20 min and stained with 0.1% crystal violet in distilled water for 15 min at RT. The crystal violet dye was then dissolved using 100 μL absolute methanol per well and the optical density of the color is measured at 570 nm using Anthos Zenyth 200rt plate reader (Anthos Labtec Instruments, Heerhugowaard, Netherlands). The IC_50_ of the compound is that required to reduce the virus-induced cytopathic effect (CPE) by 50%, relative to the virus control.

### 3.5. Docking Study

In silico docking study was performed using Autodock vina [37]. The crystal structures for the investigated targets were downloaded from the RCSB protein data bank in PDB formats. The PDB codes 7LTJ, 6WX4, 5RL9, 7BV2, and 6M0J were used for M^pro^, PL^pro^, RNA helicase, RdRp, and the S1 subunit of the viral spike protein respectively. The structures of the tested molecules were drawn using ChemDraw and converted to PDB formats using Pymol software [38]. They were further prepared for the docking study and converted to PDBQT formats using Autodock tools. The protein crystal structures were prepared by removal of water molecules, repairing missing atoms, and the addition of charges using Autodock tools. The binding site coordinates were determined using a grid box around the co-crystallized ligand and the residues involved in the interaction. A grid box with the dimensions of 40 × 40 × 40 and spacing of 0.375 Å with X, Y, and Z coordinates of −11.645, 16.822, 69.255; 10.839, −24.477, −36.709; −13.52, 38.847, −23.29; 93.304, 89.401, 100.097; −37.272, 16.941, 6.246; were used for M^pro^, PL^pro^, RNA helicase, RdRp, and viral spike protein respectively. Docking scores of different obtained modes are represented in Table S1. The obtained docking poses with the least RMSD values for the most active compound were visualized using Pymol [38]. The 2D plots of the protein-ligand interactions were obtained by LigPlot^+^ (Figure S9).

### 3.6. Molecular Dynamic Simulation and Binding Free Energy Calculation

The binding free energy calculation (∆*G*) and molecular dynamic simulation were carried out as previously described [39]. The Supplementary Materials include a detailed description of these methods.

## 4. Conclusions

After extensive chromatographic isolation, the naphthopyrone derivative, rubasperone B (**6**), was recovered for the first time from the fermentation broth of marine-derived *A. niger* along with four previously isolated naphthopyrones: flavasperone (**1**), rubrofusarin B (**2**), aurasperone A (**3**), fonsecinone A (**4**), and an alkaloid called aspernigrin A (**5**). All of these fungal compounds were screened for their inhibitory activity against SARS CoV-2 in vitro, whereby aurasperone A (**3**) was found to have the highest inhibitory activity and the lowest cytotoxic activity. Accordingly, it was subjected to docking-based virtual screening against several suggested SARS CoV-2 protein structures to find out which is/are the probable target(s) of this compound. All proposed targets showed considerable docking scores with this compound, particularly, M^pro^ which got the best score. Subsequent MDS-based screening revealed that aurasperone A (**3**) has a very good affinity towards M^pro^’s active site, and it can achieve stable binding with it over 150 ns establishing multiple H-bonds, water bridges, and hydrophobic interactions. Our findings in the present study might be a very good starting point to develop more potent anti-SARS CoV-2 M^pro^ inhibitors.

## Figures and Tables

**Table 1:** Docking scores of *Aspergillus niger* secondary metabolites against the SARS-CoV-2 main therapeutic targets using AutoDock Vina.

Compound	Binding Energy (kcal/mol)				
	M^pro^	PL^pro^	Helicase	RdRp	Spike Protein
Flavasperone (**1**)	−7.1	−6.4	−7.6	−6.9	−5.8
Rubrofusarin B (**2**)	−6.9	−5.9	−7.0	−6.8	−5.8
Aurasperone A (**3**)	−8.1	−7.4	−8.0	−7.8	−7.0
Fonsecinone A (**4**)	−8.0	−7.1	−8.1	−8.2	−7.1
Aspernigrin A (**5**)	−6.2	−6.3	−6.7	−6.5	−5.9
Rubasperone B (**6**)	−8.5	−6.8	−8.0	−7.9	−7.0
Reference inhibitor *	−7.5	−6.7	−5.6	−6.6	-

* The used reference inhibitors are N3 peptide inhibitor for M^pro^ [33], VIR251 peptide inhibitor for PL^pro^ [34], 1-(3-fluoro-4-methylphenyl)methanesulfonamide for Helicase [35], and triphosphate form of Remdesivir (RTP) for RdRp [31].

## Data Availability

Not applicable.

## Supplementary Materials

The following are available online at https://www.mdpi.com/article/10.3390/md20030179/s1, Figure S1: ESI^+^ mass spectrum of compound **6**, Figures S2–S8: NMR spectra of compound **6**, Figure S9: 2D plots of the most active compound (aurasperone A (3)) binding within the active site of: A. M^pro^; B. PL^pro^; C. RNA helicase; D. RdRp; E. viral spike protein, showing the amino acid residues involved in the interaction. Table S1: Docking scores of different modes of the tested *Aspergillus niger* secondary metabolites against the SARS-CoV-2 main therapeutic targets using AutoDock Vina.
