# Supplementary material for: Aurasperone A Inhibits SARS CoV-2 In Vitro: An Integrated In Vitro and In Silico Study
Source: Mar Drugs. 2022 Feb 28;20(3):179. doi: 10.3390/md20030179 (PMC8949533; doi:10.3390/md20030179)
Supplement: Supplementary file 1 [file marinedrugs-20-00179-s001.zip › marinedrugs-1594414 supp final/marinedrugs-1594414 supp final.pdf]

Supplementary Information

Aurasperone A inhibits SARS CoV-2 in-vitro: an integrated in-vitro and in-silico study

Mai H. ElNaggar[1,*], Ghada M. Abdelwahab[2, 3, *], Omnia Kutkat[4], Mohamed GabAllah[4], Mohamed A. Ali[4], Mohamed E.A. El-Metwally[5], Ahmed M. Sayed[6,7], Usama Ramadan Abdelmohsen[8, 9], Ashraf Taha Khalil[2]

[1]Department of Pharmacognosy, Faculty of Pharmacy, Kafrelsheikh university, 33516, Kafrelsheikh, Egypt.

[2]Department of Pharmacognosy, Faculty of Pharmacy, Mansoura University, 35516, Mansoura, Egypt.

[3]Department of Pharmacognosy, Faculty of Pharmacy, Horus University, 34518, Damietta, Egypt.

[4]Center of Scientific Excellence for Influenza Viruses, National Research Centre, Giza 12622, Egypt.

[5]National Institute of Oceanography and Fisheries (NIOF), Egypt.

[6]Department of Pharmacognosy, Faculty of Pharmacy, Nahda University, 62513 Beni-Suef, Egypt.

[7]Department of Pharmacognosy, Faculty of Pharmacy, Almaaqal University, 61014 Basra, Iraq.

[8]Department of Pharmacognosy, Faculty of Pharmacy, Minia University, 61519 Minia, Egypt

[9]Department of Pharmacognosy, Faculty of Pharmacy, Deraya University, 61111 New Minia, Egypt

*Authors to whom correspondence should be addressed

1. Characterization of compound 6

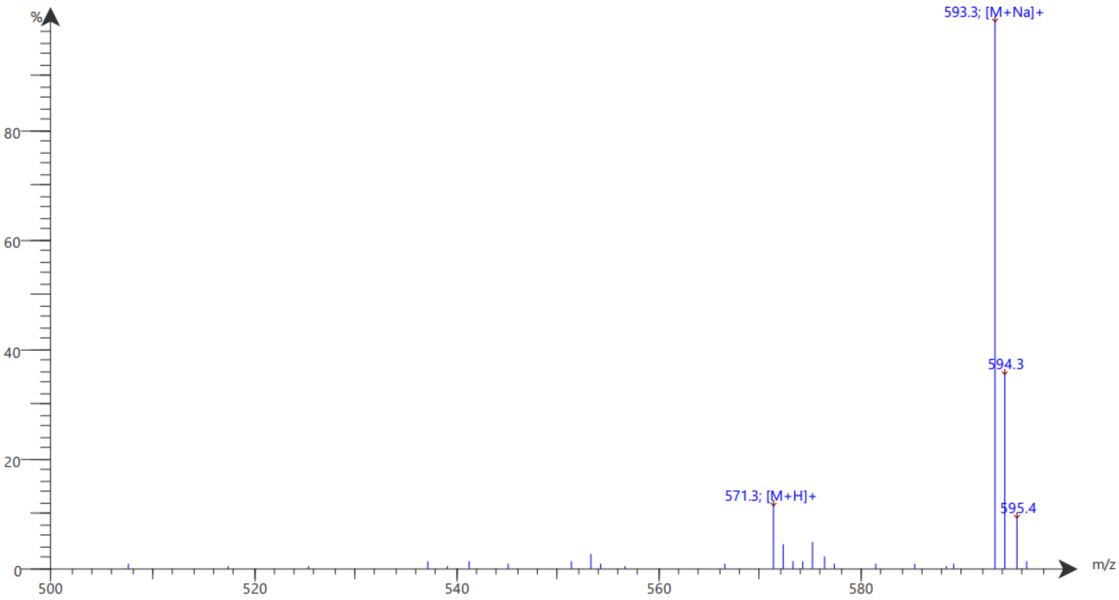

Figure S1: ESI$^+$ mass spectrum of compound **6 (**Rubasperone B)

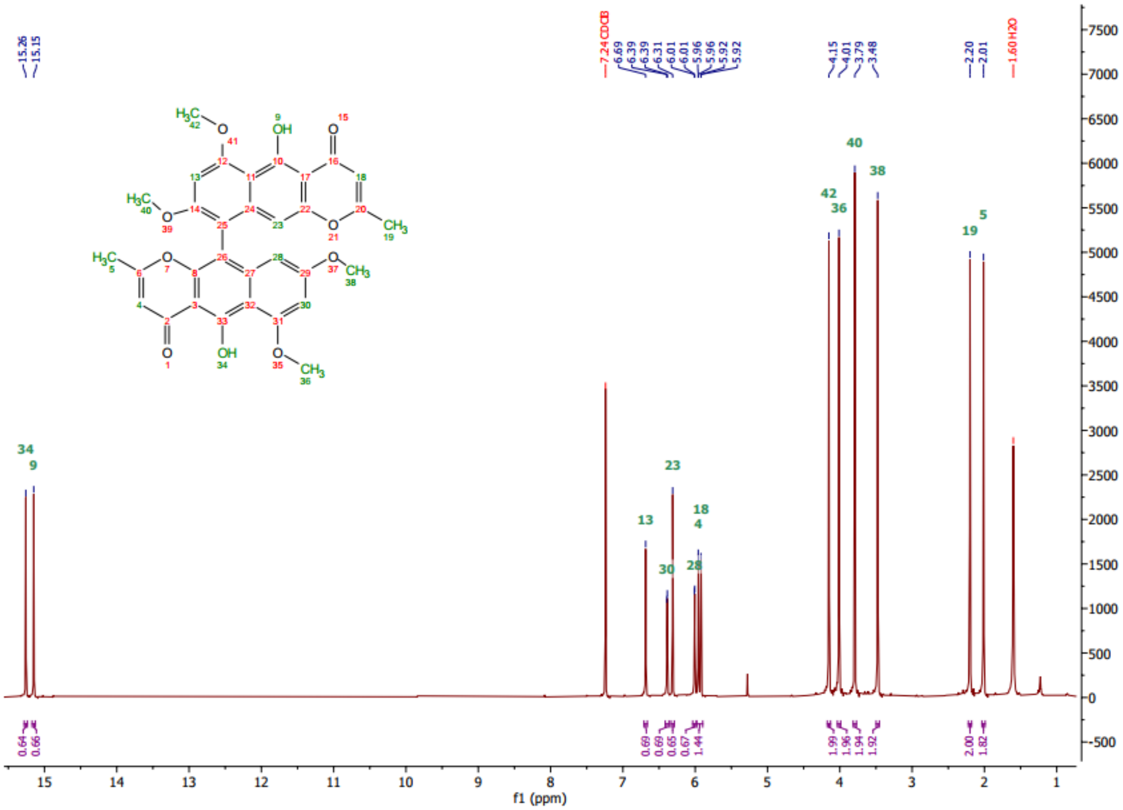

Figure S2: ^{1}H-NMR spectrum of compound **6 (**Rubasperone B)

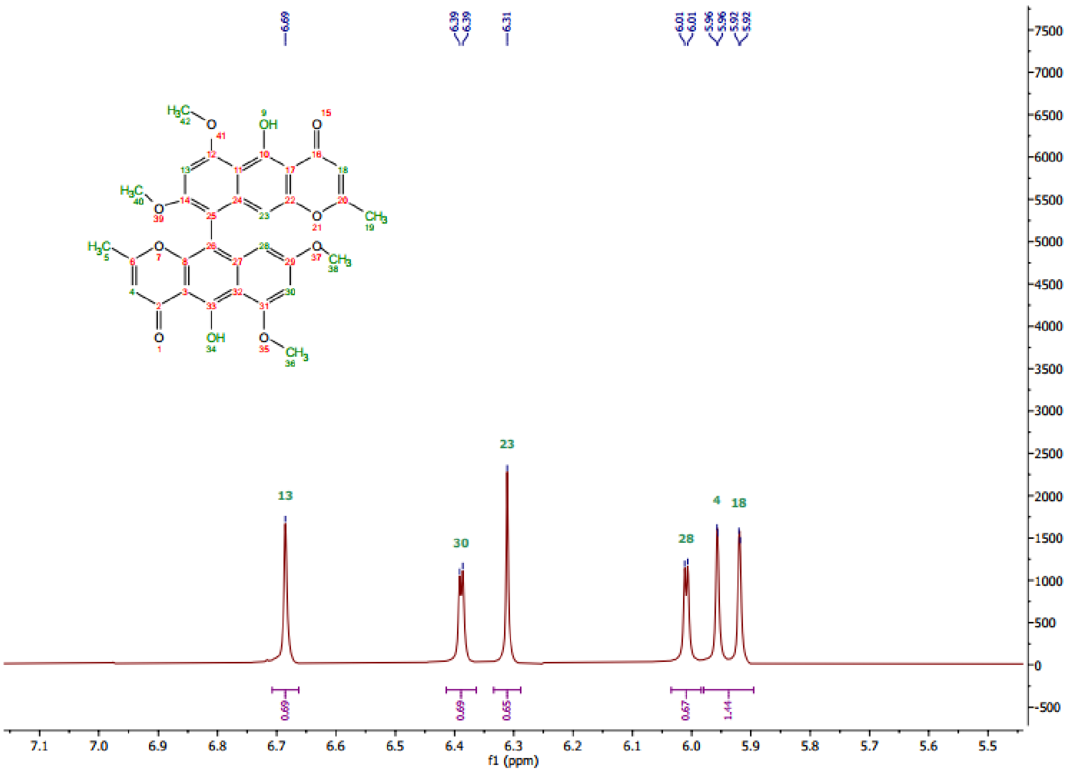

Figure S3: Expanded ^{1}H-NMR spectrum of compound **6 (**Rubasperone B)

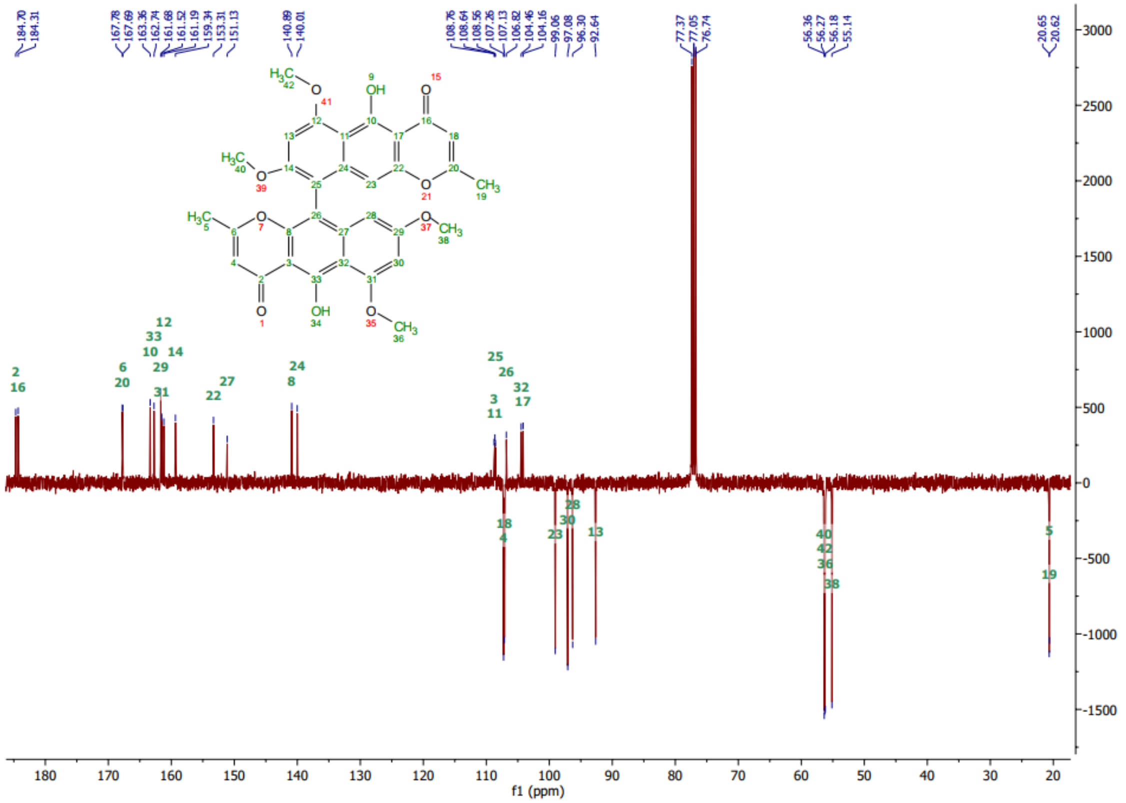

Figure S4: APT spectrum of compound **6 (**Rubasperone B)

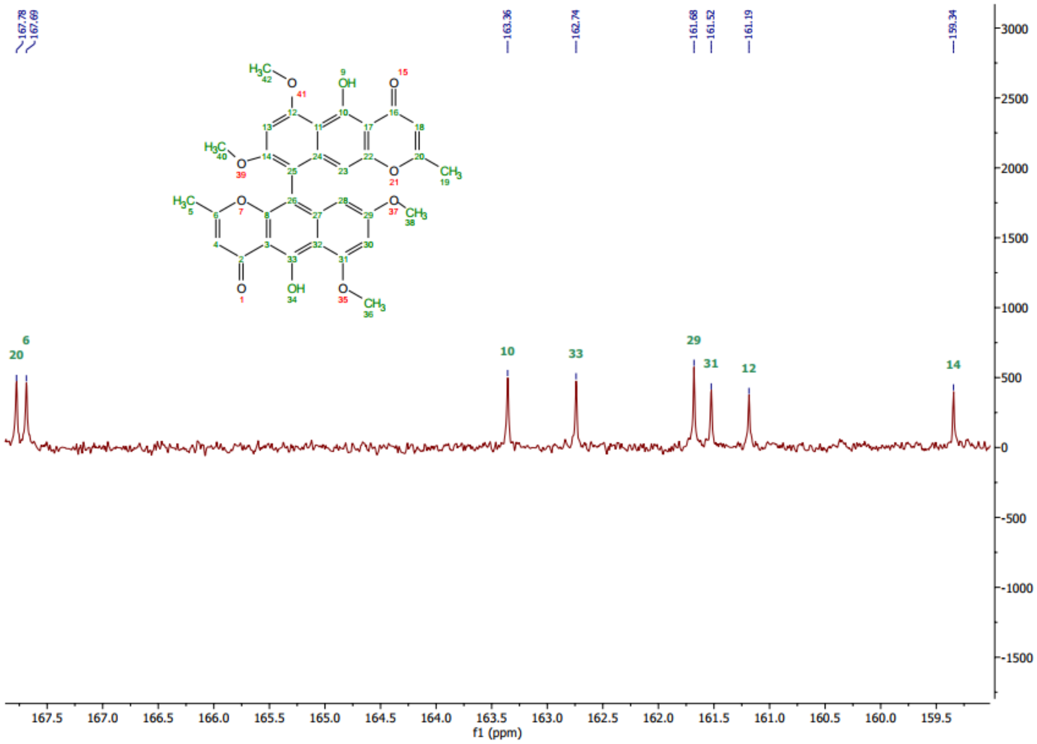

Figure S5: Expanded APT spectrum of **compound 6 (**Rubasperone B)

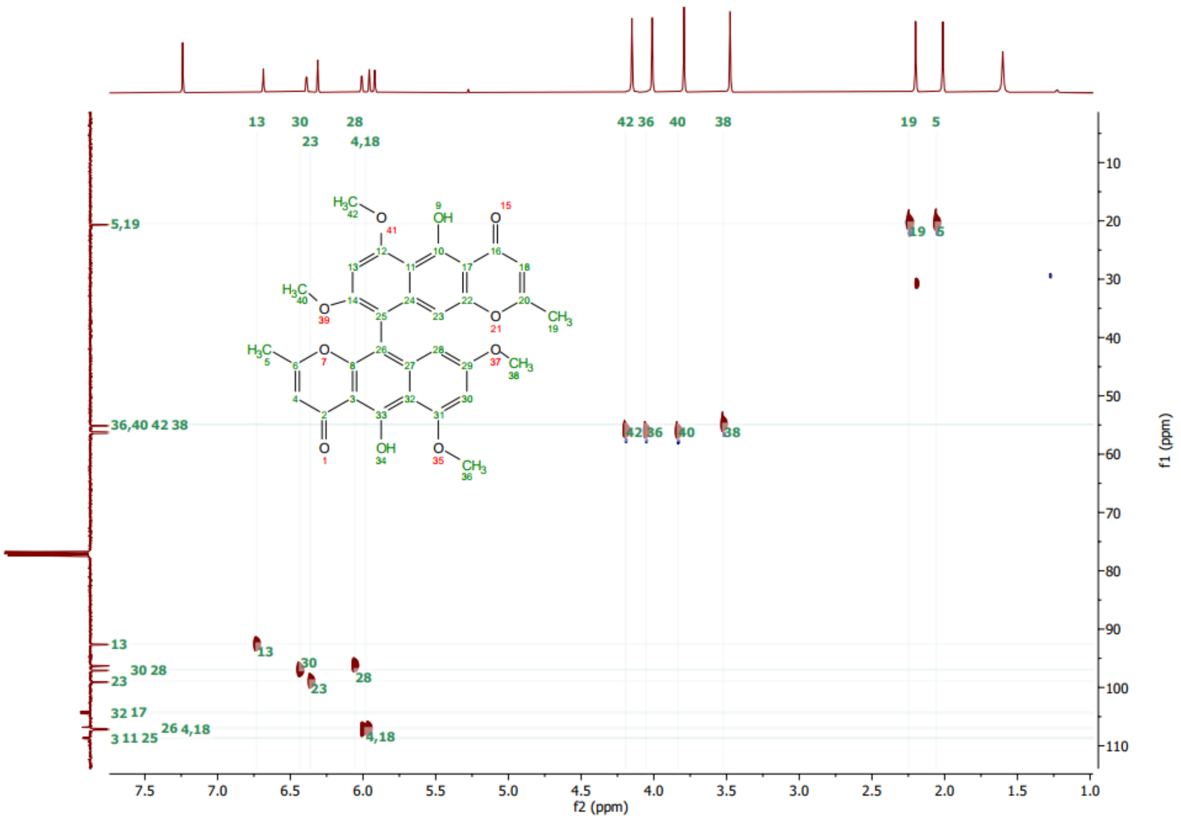

Figure S6: HSQC spectrum of compound **6 (**Rubasperone B)

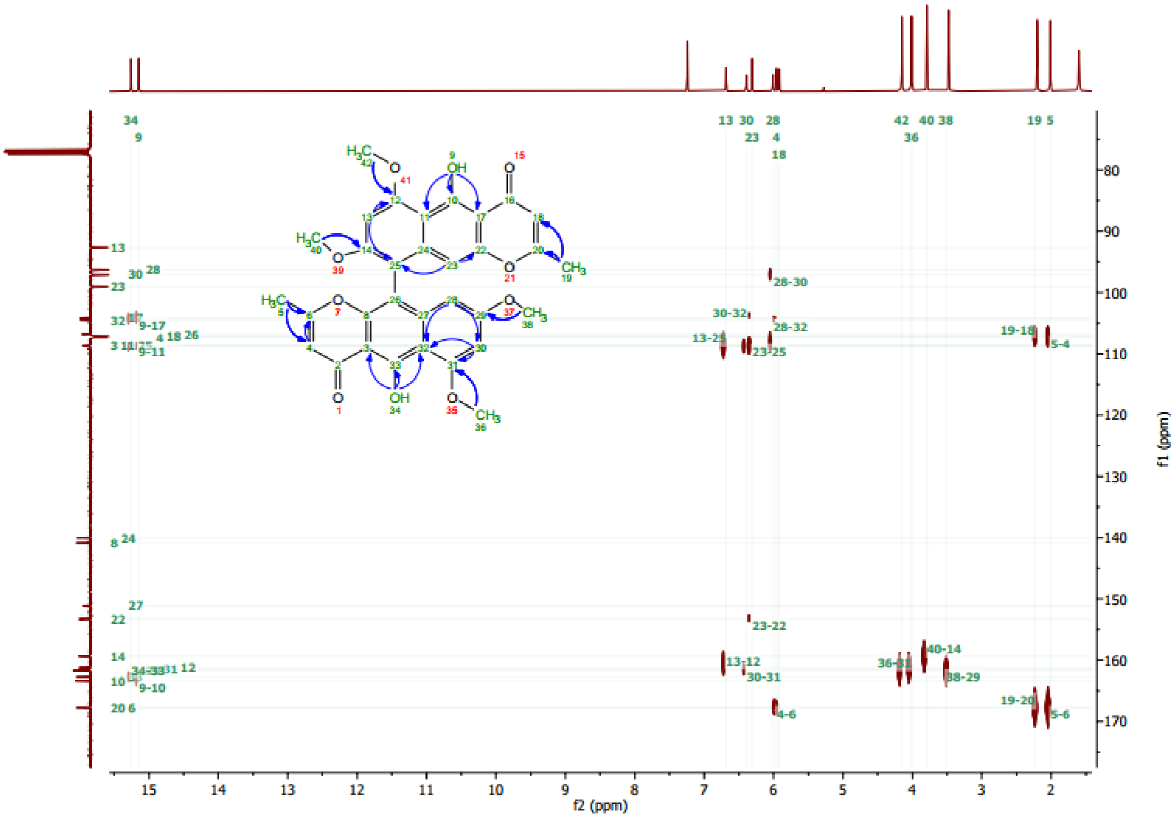

Figure S7: HMBC spectrum of compound **6 (**Rubasperone B)

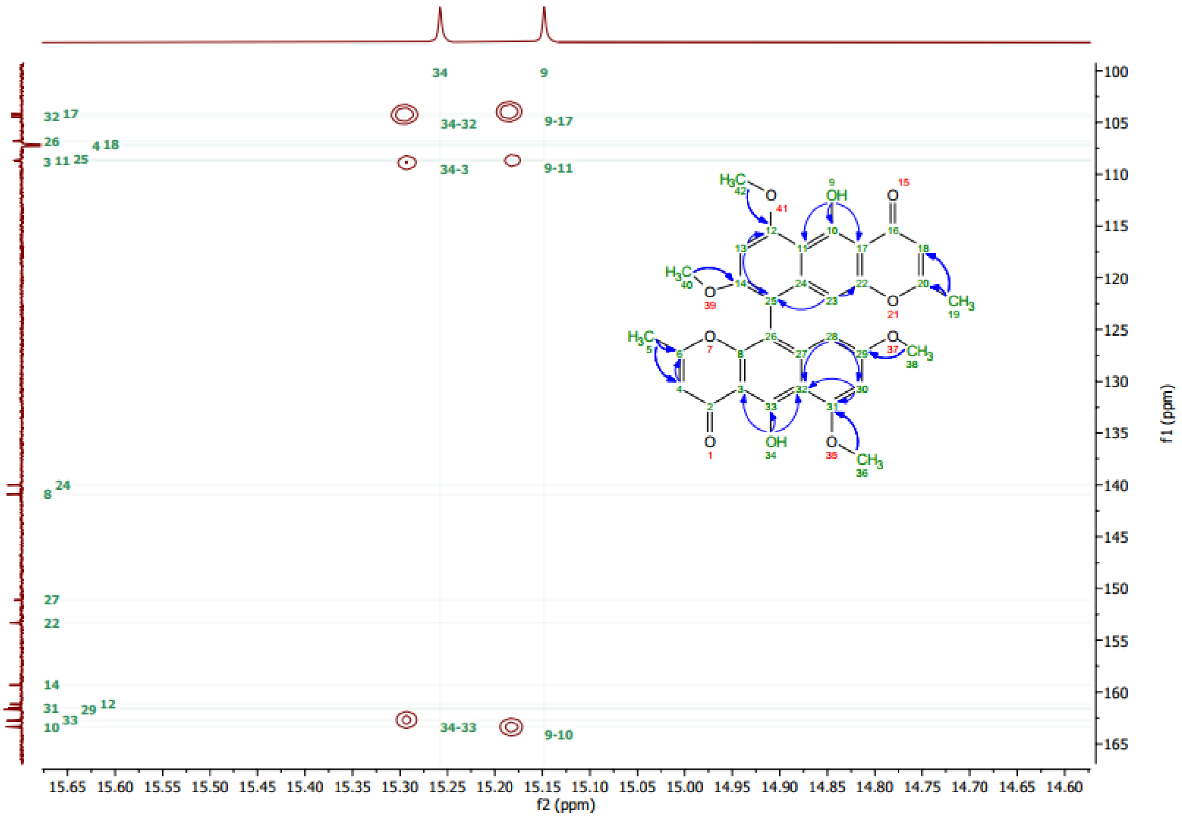

Figure S8: Expanded HMBC spectrum of compound **6 (**Rubasperone B)

2. **Docking study**

Table S1. Docking scores of different modes of the tested *Aspergillus niger* secondary metabolites against the SARS-CoV-2 main therapeutic targets using AutoDock Vina.

**Compound**	M^{pro}				PL^{pro}			
	mode	affinity (kcal/mol)	dist from best mode rmsd l.b.	dist from best mode rmsd u.b.	mode	affinity (kcal/mol)	dist from best mode rmsd l.b.	dist from best mode rmsd u.b.
**Flavasperone (1)**	1	-7.1	0.000	0.000	1	-6.4	0.000	0.000
	2	-6.6	2.231	5.387	2	-5.9	17.761	20.243
	3	-6.6	1.294	3.710	3	-5.9	2.113	5.352
	4	-6.5	1.689	3.875	4	-5.8	1.838	2.760
	5	-6.5	4.044	5.294	5	-5.8	17.779	20.268
	6	-6.4	1.485	2.482	6	-5.7	19.124	21.735
	7	-6.3	2.651	4.350	7	-5.4	1.898	5.806
	8	-6.0	2.970	5.295	8	-5.4	2.376	5.709
	9	-6.0	2.650	7.324	9	-5.4	12.201	14.197
	10	-5.9	2.412	5.818	10	-5.3	19.105	21.772
**Rubrofusarin B (2)**	1	-6.9	0.000	0.000	1	-5.9	0.000	0.000
	2	-6.6	2.281	4.247	2	-5.7	14.572	16.105
	3	-6.3	2.247	4.276	3	-5.7	0.988	5.964
	4	-6.3	2.116	5.631	4	-5.7	1.403	6.169
	5	-6.1	3.099	4.797	5	-5.6	13.230	16.328
	6	-6.1	2.646	4.732	6	-5.6	12.611	15.417
	7	-6.1	3.581	6.627	7	-5.6	12.195	14.620
	8	-6.0	2.617	3.078	8	-5.5	2.238	4.799
	9	-5.9	2.248	4.527	9	-5.5	12.691	14.529
	10	-5.9	15.316	17.291	10	-5.4	11.768	13.897

Compound	mode	affinity (kcal/mol)	dist from best mode rmsd l.b.	dist from best mode rmsd u.b.	mode	affinity (kcal/mol)	dist from best mode rmsd l.b.	dist from best mode rmsd u.b.
**Aurasperone A (3)**	1	-8.1	0.000	0.000	1	-7.4	0.000	0.000
	2	-8.1	2.111	8.838	2	-7.3	3.505	5.716
	3	-7.8	2.067	8.569	3	-6.9	0.828	4.202
	4	-7.5	3.261	7.107	4	-6.7	2.991	5.455
	5	-7.4	3.168	5.677	5	-6.6	3.230	8.285
	6	-7.4	3.340	7.589	6	-6.6	2.942	6.901
	7	-7.1	2.942	5.224	7	-6.4	4.049	6.192
	8	-7.1	2.792	7.130	8	-6.2	9.927	15.348
	9	-7.0	16.102	19.379	9	-6.2	3.119	6.988
	10	-6.6	4.166	8.987	10	-6.2	3.294	4.205
**Fonsecinone A (4)**	1	-8.5	0.000	0.000	1	-7.1	0.000	0.000
	2	-8.0	1.054	4.216	2	-6.9	2.595	6.628
	3	-7.6	3.221	6.325	3	-6.8	2.742	5.963
	4	-7.5	2.024	8.383	4	-6.7	3.228	8.051
	5	-7.1	5.070	9.583	5	-6.7	1.092	4.198
	6	-7.1	2.638	6.338	6	-6.6	3.595	6.780
	7	-7.0	3.835	7.213	7	-6.5	3.826	6.164
	8	-6.7	2.333	8.417	8	-6.3	2.138	4.246
	9	-6.6	4.033	7.799	9	-6.1	2.437	7.614
	10	-6.4	2.972	6.215	10	-6.1	2.536	5.854

Compound	mode	affinity (kcal/mol)	dist from best mode rmsd l.b.	dist from best mode rmsd u.b.	mode	affinity (kcal/mol)	dist from best mode rmsd l.b.	dist from best mode rmsd u.b.
Aspernigrin A (5)	1	-6.2	0.000	0.000	1	-6.3	0.000	0.000
	2	-6.1	3.331	6.314	2	-6.1	2.842	3.914
	3	-6.1	2.051	2.719	3	-6.0	3.520	5.996
	4	-6.1	1.648	2.154	4	-5.8	4.555	6.053
	5	-6.0	4.186	5.386	5	-5.8	2.954	6.139
	6	-5.8	1.724	2.235	6	-5.7	2.629	6.604
	7	-5.8	4.008	6.227	7	-5.6	2.858	6.858
	8	-5.7	3.899	5.245	8	-5.4	2.769	6.340
	9	-5.6	3.273	4.566	9	-5.1	3.870	8.526
	10	-5.5	16.135	17.886	10	-5.1	12.465	14.959
Rubasperone B (6)	1	-8.5	0.000	0.000	1	-6.8	0.000	0.000
	2	-8.2	1.534	7.601	2	-6.4	10.760	14.566
	3	-8.0	0.917	4.222	3	-6.4	10.255	14.248
	4	-7.2	2.318	5.398	4	-6.3	11.438	16.454
	5	-7.2	2.507	5.546	5	-6.3	11.599	15.282
	6	-7.1	2.525	6.626	6	-6.2	9.957	15.077
	7	-7.1	1.692	7.627	7	-6.1	10.335	14.933
	8	-7.1	2.344	5.022	8	-6.1	10.955	14.434
	9	-7.0	2.427	7.098	9	-6.1	11.210	16.876
	10	-6.8	2.280	7.784	10	-6.0	8.301	12.911

	mode	affinity (kcal/mol)	dist from best mode rmsd l.b.	dist from best mode rmsd u.b.	mode	affinity (kcal/mol)	dist from best mode rmsd l.b.	dist from best mode rmsd u.b.
Reference inhibitor *	1	-7.5	0.000	0.000	1	-6.7	0.000	0.000
	2	-7.3	1.508	2.119	2	-6.1	2.898	4.545
	3	-7.2	2.609	7.639	3	-6.1	2.107	2.846
	4	-7.2	3.889	7.746	4	-5.8	2.513	3.542
	5	-7.2	3.101	7.602	5	-5.7	2.924	7.823
	6	-7.2	2.219	7.211	6	-5.7	2.479	7.621
	7	-7.2	2.412	6.872	7	-5.7	2.730	5.295
	8	-7.0	2.028	7.396	8	-5.7	2.743	4.245
	9	-6.8	2.813	5.776	9	-5.7	2.693	5.104
	10	-6.8	2.805	8.289	10	-5.6	19.595	23.575

Compound	mode	Helicase affinity (kcal/mol)	Helicase dist from best mode rmsd l.b.	Helicase dist from best mode rmsd u.b.	RdRp affinity (kcal/mol)	RdRp dist from best mode rmsd l.b.	RdRp dist from best mode rmsd u.b.
Flavasperone (1)	1	-7.6	0.000	0.000	-6.9	0.000	0.000
	2	-7.1	2.138	6.313	-6.3	21.429	26.697
	3	-6.6	1.902	5.095	-6.0	11.156	14.531
	4	-6.5	2.826	5.242	-6.0	23.537	26.194
	5	-6.5	1.641	5.606	-6.0	22.754	26.133
	6	-6.5	3.340	4.408	-6.0	22.161	25.421
	7	-6.4	3.124	5.393	-6.0	22.153	25.967
	8	-6.4	1.716	3.717	-5.9	24.513	27.463
	9	-6.3	3.519	5.993	-5.8	2.616	4.822
	10	-6.2	2.880	5.209	-5.8	22.732	25.849
Rubrofusarin B (2)	1	-7.0	0.000	0.000	-6.8	0.000	0.000
	2	-6.8	1.665	3.684	-6.3	24.457	28.576
	3	-6.7	3.312	6.020	-6.2	26.828	29.390
	4	-6.7	1.696	3.528	-6.2	2.057	4.741
	5	-6.6	2.496	3.868	-6.0	26.933	28.414
	6	-6.6	1.916	6.692	-6.0	25.898	28.686
	7	-6.5	2.065	3.810	-5.8	23.027	26.744
	8	-6.3	8.228	12.363	-5.8	23.261	27.651
	9	-6.3	3.926	5.780	-5.7	24.368	27.564
	10	-6.2	2.336	6.455	-5.7	4.992	8.055

	mode	affinity (kcal/mol)	dist from best mode rmsd l.b.	dist from best mode rmsd u.b.	mode	affinity (kcal/mol)	dist from best mode rmsd l.b.	dist from best mode rmsd u.b.
Aurasperone A (3)	1	-8.0	0.000	0.000	1	-7.8	0.000	0.000
	2	-7.7	0.798	4.214	2	-7.7	2.589	8.664
	3	-7.4	2.632	6.858	3	-7.5	2.449	8.013
	4	-7.3	2.345	8.520	4	-7.5	2.826	6.008
	5	-7.0	3.283	4.882	5	-7.4	2.769	9.781
	6	-7.0	2.426	8.861	6	-7.4	2.554	3.889
	7	-6.9	2.509	6.477	7	-7.4	11.542	15.763
	8	-6.8	2.498	8.021	8	-7.3	3.475	9.212
	9	-6.7	2.951	5.834	9	-7.2	4.225	8.543
	10	-6.7	2.550	6.257	10	-7.1	3.053	5.036
Fonsecinone A (4)	1	-8.1	0.000	0.000	1	-8.2	0.000	0.000
	2	-7.8	3.163	8.579	2	-8.0	1.329	4.415
	3	-7.6	10.553	14.851	3	-7.8	9.301	13.699
	4	-7.6	13.958	18.707	4	-7.7	25.228	29.473
	5	-7.5	12.482	16.428	5	-7.7	22.936	28.551
	6	-7.3	13.809	18.932	6	-7.7	23.984	29.211
	7	-7.2	12.804	17.450	7	-7.6	25.633	28.818
	8	-7.1	14.581	18.306	8	-7.5	25.924	29.895
	9	-7.1	2.520	5.600	9	-7.4	22.320	28.017
	10	-7.0	13.017	17.499	10	-7.3	7.853	12.962

Compound	mode	affinity (kcal/mol)	dist from best mode rmsd l.b.	rmsd u.b.	mode	affinity (kcal/mol)	dist from best mode rmsd l.b.	rmsd u.b.
**Aspernigrin A (5)**	1	-6.7	0.000	0.000	1	-6.5	0.000	0.000
	2	-6.7	3.257	4.122	2	-6.4	10.658	11.724
	3	-6.6	1.382	2.256	3	-6.3	10.763	12.893
	4	-6.6	3.857	6.217	4	-6.1	13.179	14.281
	5	-6.5	4.619	6.679	5	-6.1	17.227	18.794
	6	-6.3	3.821	5.068	6	-6.1	15.115	16.205
	7	-6.2	4.260	6.464	7	-5.9	12.133	13.260
	8	-5.9	3.716	6.381	8	-5.8	12.810	13.810
	9	-5.9	4.299	6.361	9	-5.8	15.240	16.736
	10	-5.8	8.103	10.154	10	-5.8	10.881	11.855
**Rubasperone B (6)**	1	-8.0	0.000	0.000	1	-7.9	0.000	0.000
	2	-8.0	1.399	7.722	2	-7.9	20.483	24.573
	3	-7.4	22.483	27.715	3	-7.7	7.246	11.382
	4	-7.4	1.284	7.571	4	-7.6	7.063	11.223
	5	-7.4	7.213	10.523	5	-7.6	19.189	23.222
	6	-7.2	20.221	25.155	6	-7.6	1.199	7.396
	7	-7.2	1.780	4.884	7	-7.6	20.569	24.294
	8	-7.1	17.225	22.425	8	-7.5	16.469	21.113
	9	-6.9	21.880	26.641	9	-7.5	15.575	20.480
	10	-6.9	17.043	21.971	10	-7.4	1.015	4.313

	mode	affinity (kcal/mol)	dist from best mode rmsd l.b.	rmsd u.b.	mode	affinity (kcal/mol)	dist from best mode rmsd l.b.	rmsd u.b.
	1	-5.6	0.000	0.000	1	-6.6	0.000	0.000
	2	-5.6	1.327	1.875	2	-6.3	5.397	8.392
	3	-5.5	1.734	2.240	3	-6.2	22.787	24.573
**Reference inhibitor** *	4	-5.3	5.191	6.949	4	-6.1	4.926	7.054
	5	-5.2	4.914	6.909	5	-5.8	5.815	8.191
	6	-5.2	2.439	3.066	6	-5.7	1.970	2.516
	7	-5.2	2.345	2.985	7	-5.7	1.767	2.708
	8	-5.0	11.659	12.890	8	-5.6	4.653	7.073
	9	-5.0	2.139	2.632	9	-5.6	19.973	22.022
	10	-4.8	3.730	4.446	10	-5.5	4.669	7.025

Spike protein

Flavasperone (1)

mode	affinity (kcal/mol)	dist from best mode rmsd l.b.	rmsd u.b.
1	-5.8	0.000	0.000
2	-5.6	3.567	6.478
3	-5.5	14.620	16.535
4	-5.5	7.673	12.049
5	-5.4	17.906	20.161
6	-5.4	3.608	6.019
7	-5.4	13.999	16.229
8	-5.4	17.583	19.559
9	-5.4	16.691	19.355
10	-5.2	15.770	18.059

Rubrofusarin B (2)

mode	affinity (kcal/mol)	dist from best mode rmsd l.b.	rmsd u.b.
1	-5.8	0.000	0.000
2	-5.6	18.613	21.431
3	-5.6	18.397	21.710
4	-5.6	17.680	20.384
5	-5.6	16.972	19.089
6	-5.5	2.528	6.742
7	-5.4	2.925	7.388
8	-5.3	18.754	21.794
9	-5.3	17.672	21.625
10	-5.3	18.496	22.115

Aurasperone A (3)

mode	affinity (kcal/mol)	dist from best mode rmsd l.b.	rmsd u.b.
1	-7.0	0.000	0.000
2	-6.8	1.306	2.776
3	-6.6	2.733	7.252
4	-6.3	11.151	16.172
5	-6.3	3.589	5.662
6	-6.3	2.025	5.708
7	-6.2	4.087	6.164
8	-6.1	1.792	5.097
9	-5.9	16.705	20.521
10	-5.9	6.938	14.178

Fonsecinone A (4)

mode	affinity (kcal/mol)	dist from best mode rmsd l.b.	rmsd u.b.
1	-7.1	0.000	0.000
2	-6.8	2.697	8.201
3	-6.8	3.821	9.148
4	-6.3	0.924	4.349
5	-6.3	3.822	9.597
6	-6.3	3.825	10.873
7	-6.3	4.851	9.724
8	-6.3	21.666	26.291
9	-6.2	4.801	8.786
10	-6.2	3.765	6.623

Aspernigrin A (5)

mode	affinity (kcal/mol)	dist from best mode rmsd l.b.	rmsd u.b.
1	-5.9	0.000	0.000
2	-5.8	16.990	18.120
3	-5.8	22.004	23.557
4	-5.8	1.075	1.996
5	-5.6	22.261	23.780
6	-5.5	16.056	17.331
7	-5.3	22.120	23.529
8	-5.2	12.286	14.708
9	-5.1	17.421	19.098
10	-5.1	22.748	24.661

Rubasperone B (6)

mode	affinity (kcal/mol)	dist from best mode rmsd l.b.	rmsd u.b.
1	-7.0	0.000	0.000
2	-6.8	1.450	7.461
3	-6.7	20.708	25.292
4	-6.7	2.118	7.788
5	-6.5	2.491	5.756
6	-6.3	1.621	7.422
7	-6.2	2.818	5.650
8	-6.1	21.286	25.551
9	-6.1	2.561	6.912
10	-6.1	2.627	5.206

A.

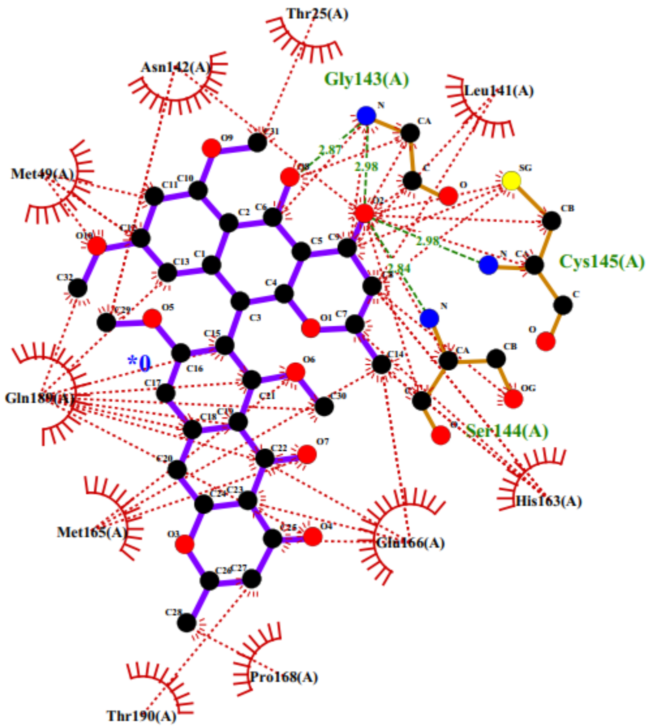

Thr25(A)
Asn142(A)
Gly143(A)
Leu141(A)
Met49(A)
Cys145(A)
Gln189(A)
Ser144(A)
His163(A)
Met165(A)
Glu166(A)
Pro168(A)
Thr190(A)

B.

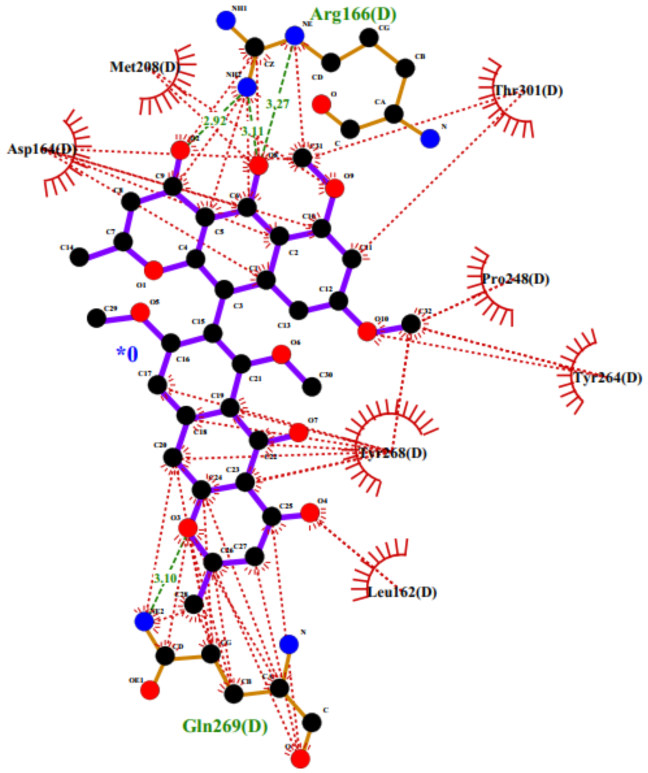

Arg166(D)
Met208(D)
Thr301(D)
Asp164(D)
Pro248(D)
Tyr264(D)
Tyr268(D)
Leu162(D)
Gln269(D)

C.

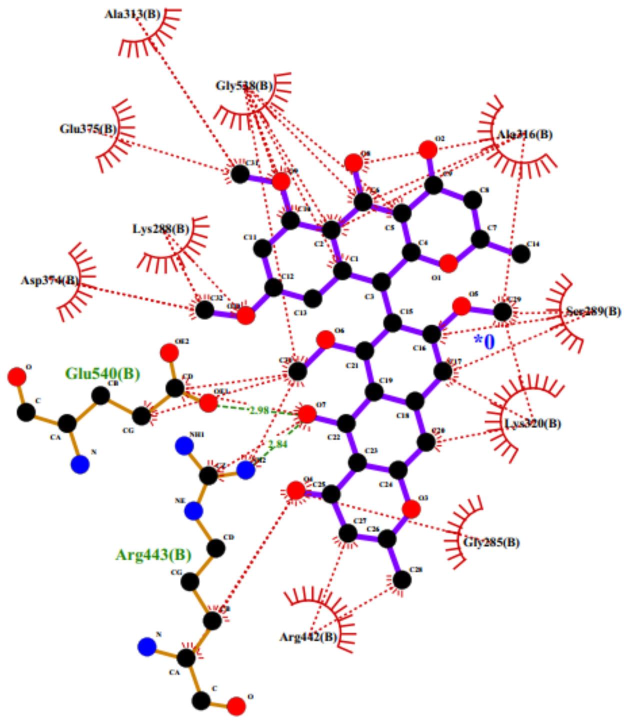

Ala313(B)
Gly538(B)
Glu375(B)
Ala316(B)
Lys288(B)
Asp374(B)
Ser289(B)
Glu540(B)
Lys320(B)
Arg443(B)
Gly285(B)
Arg442(B)

D.

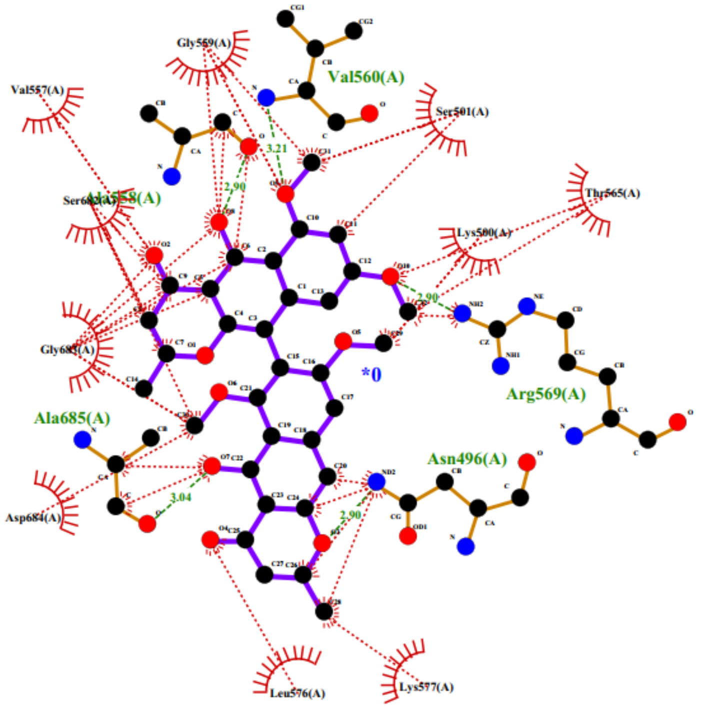

Gly559(A)
Val560(A)
Val557(A)
Ser501(A)
Thr565(A)
Lys580(A)
Arg569(A)
Ala685(A)
Asn496(A)
Asp684(A)
Leu576(A)
Lys577(A)

E.

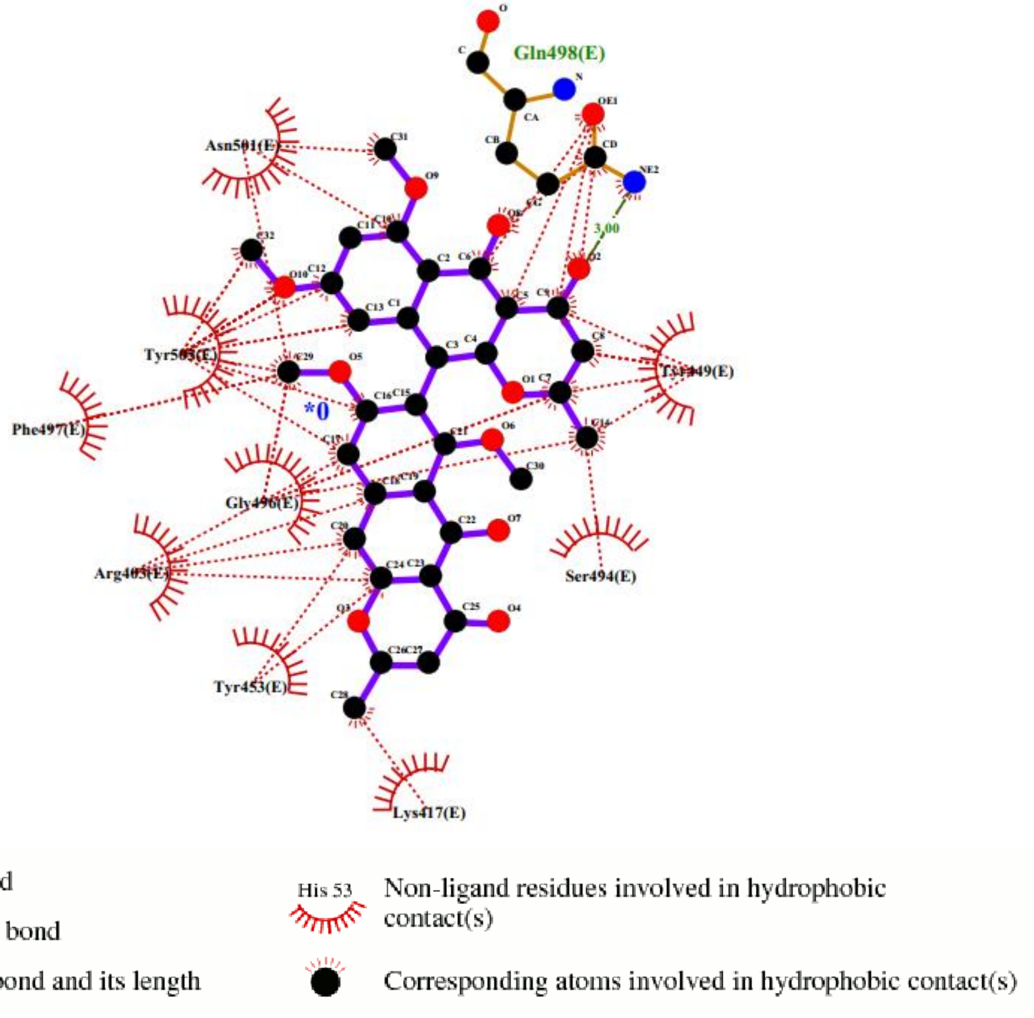

Figure S9. 2D plots of the most active compound (Aurasperone A (**3**)) binding within the active site of: A. M^{pro}; B. PL^{pro}; C. RNA helicase; D. RdRp; E. viral spike protein, showing the amino acid residues involved in the interaction.

3. Molecular Dynamics Simulation

Desmond v. 2.2 software was used for performing MDS experiments [1–3]. This software applies the OPLS-2005 force field. Protein systems were built using the System Builder option, where the protein structure was checked for any missing hydrogens, the protonation states of the amino acid residues were set (pH = 7.4), and the co-crystalized water molecules were removed. Thereafter, the whole structure was embedded in an orthorhombic box of TIP3P water together with 0.15 M Na^+ and Cl^- ions in 20 Å solvent

buffer. Afterward, the prepared systems were energy minimized and equilibrated for 10 ns. For protein-ligand complexes, the top-scoring poses were used as a starting points for simulation. Desmond software automatically parameterizes inputted ligands during the system building step according to the OPLS force field. For simulations performed by NAMD [4], the protein structures were built and optimized by using the QwikMD toolkit of the VMD software. The parameters and topologies of the compounds were calculated using the Charmm27 force field with the online software Ligand Reader and Modeler (http://www.charmm-gui. org/?doc=input/ligandrm, accessed on 16 April 2021) [5]. Afterward, the generated parameters and topology files were loaded to VMD to readily read the protein–ligand complexes without errors and then conduct the simulation step.

4. Binding Free Energy Calculations

Binding free energy calculations (Δ*G*) were performed using the free energy perturbation (FEP) method [4]. This method was described in detail in the recent article by Kim and coworkers [4]. Briefly, this method calculates the binding free energy Δ*G*binding according to the following equation: $\Delta G_{binding} = \Delta G_{Complex} - \Delta G_{Ligand}$. The value of each Δ*G* is estimated from a separate simulation using NAMD software. Interestingly, all input files required for simulation by NAMD can be papered by using the online website CharmmGUI (https://charmm-gui.org/?doc=input/afes.abinding). Subsequently, we can use these files in NAMD to produce the required simulations using the FEP calculation function in NAMD. The equilibration was achieved in the NPT ensemble at 300 K and 1 atm (1.01325 bar) with Langevin piston pressure (for “Complex” and “Ligand”) in the presence of the TIP3P water model. Then, 10 ns FEP simulations were performed for each compound, and the last 5 ns of the free energy values was measured for the final free energy values [4]. Finally, the generated trajectories were visualized and analyzed using VMD software. It worth noting that Ngo and co-workers in their recent benchmarking study found that the FEP method of determination of Δ*G* was the most accurate method in terms of predicting M^{Pro} inhibitors [5].

References

1. Bowers, K. J.; Chow, D. E.; Xu, H.; Dror, R. O.; Eastwood, M. P.; Gregersen, B. A.; Klepeis, J. L.; Kolossvary, I.; Moraes, M. A.; Sacerdoti, F. D. In *Scalable algorithms for molecular dynamics simulations on commodity clusters*, SC'06: Proceedings of the 2006 ACM/IEEE Conference on Supercomputing, 2006; IEEE: pp 43-43.
2. Release, S., 4: Desmond Molecular Dynamics System, DE Shaw Research, New York, NY. *Maestro-Desmond Interoperability Tools. Schrodinger, LLC, New York, NY* **2017**.
3. Phillips, J. C.; Braun, R.; Wang, W.; Gumbart, J.; Tajkhorshid, E.; Villa, E.; Chipot, C.; Skeel, R. D.; Kale, L.; Schulten, K., Scalable molecular dynamics with NAMD. *Journal of computational chemistry* **2005,** 26, (16), 1781-1802.
4. Kim, S.; Oshima, H.; Zhang, H.; Kern, N. R.; Re, S.; Lee, J.; Roux, B.; Sugita, Y.; Jiang, W.; Im, W., CHARMM-GUI free energy calculator for absolute and relative ligand solvation and binding free energy simulations. *Journal of chemical theory and computation* **2020,** 16, (11), 7207-7218.
5. Ngo, S. T.; Tam, N. M.; Pham, M. Q.; Nguyen, T. H., Benchmark of Popular Free Energy Approaches Revealing the Inhibitors Binding to SARS-CoV-2 Mpro. *Journal of chemical information and modeling* **2021,** 61, (5), 2302-2312.
